# Education and Alzheimer Disease Genetic Risk in Associations of GLP-1 Receptor Agonists With Dementia Among Adults With Type 2 Diabetes

**DOI:** 10.64898/2026.09.04.26362304

**Published:** 2026-09-08

**Authors:** Jingxuan Wang, Anna M. Pederson, Michael D. Flanders, Minhyuk Choi, Peter Buto, Kendra D. Sims, Ruijia Chen, Elyse Couch, Paola Gilsanz, Kaleen N. Hayes, Andrew R. Zullo, Andrew Stokes, M. Maria Glymour, Sarah F. Ackley

## Abstract

**Objective:**

To evaluate whether educational attainment and Alzheimer disease genetic risk were associated with GLP-1 receptor agonist initiation and dementia incidence and whether adjustment for these measured factors materially changed the estimated association between GLP-1 receptor agonist initiation and incident dementia among adults with type 2 diabetes.

**Research Design and Methods:**

We conducted an observational cohort study using linked electronic health record, survey, and genetic data from 14,364 All of Us Research Program participants with type 2 diabetes. We estimated associations of educational attainment and Alzheimer disease genetic risk with treatment initiation and incident dementia and compared GLP-1 receptor agonist initiators with initiators of non-sodium-glucose cotransporter 2 inhibitor second-line therapies, with a separate sodium-glucose cotransporter 2 inhibitor comparison. Models were estimated before and after additional adjustment for educational attainment, APOE ε4, and non-APOE genetic risk.

**Results:**

Among 14,364 participants (mean age, 60.2 years; 54.2% female), the mean follow-up duration was 4.3 years. The estimated hazard ratio for dementia comparing GLP-1 receptor agonist initiation with non-SGLT2 inhibitor second-line therapy was 0.85 (95% CI 0.64-1.12) before adjustment for education or Alzheimer disease genetic risk and 0.84 (95% CI 0.64-1.12) after adjustment for educational attainment, APOE ε4, and non-APOE genetic risk.

**Conclusions:**

Among adults with type 2 diabetes, adjustment for measured educational attainment and Alzheimer disease genetic susceptibility produced little change in the estimated association between GLP-1 receptor agonist initiation and incident dementia. These findings do not exclude confounding by these factors in other populations or residual confounding from socioeconomic, clinical, behavioral, and health-care-related factors.

**Highlights:**

- Observational studies have reported associations between GLP-1 receptor agonist use and lower dementia risk, but important social and genetic factors are often unavailable in electronic health record data.
- We examined whether educational attainment and Alzheimer disease genetic susceptibility were associated with treatment initiation and dementia and whether adjustment for these factors changed GLP-1 receptor agonist–dementia estimates.
- Adding measured education and genetic susceptibility produced little change in the estimated conditional hazard ratios.
- These results do not exclude residual confounding and should not be interpreted as evidence that GLP-1 receptor agonists prevent dementia.

## Full Text Availability

The license terms selected by the author(s) for this preprint version do not permit archiving in PMC. The full text is available from the preprint server.

